# Evaluation of Shear Bond Strength and Surface Morphology (via SEM) of Different Provisional Crowns and Surface Treatments to PMMA

**DOI:** 10.1155/ijod/5524320

**Published:** 2026-02-09

**Authors:** Pattarawadee Krassanairawiwong, Jirat Srihatajati

**Affiliations:** ^1^ Prosthodontics Department, College of Dental Medicine, Rangsit University, Mueang, Pathum Thani, Thailand, rsu.ac.th

**Keywords:** 3D printed, polylactic acid, polymethyl methacrylate, provisional crown, shear bond strength

## Abstract

**Purpose of the Study:**

To compare the shear bond strengths (SBSs) of different provisional crown materials with various surface treatments when relined with autopolymerizing polymethyl methacrylate (PMMA).

**Materials and Methods:**

Part 1: Seventy‐five specimens were divided into five groups: PMAC (autopolymerizing PMMA), PMAM (CAD/CAM system PMMA), PMAD (3D‐printed PMMA), PLAI (injectable polylactic acid [PLA]), and PLAD (3D‐printed PLA). All specimens were surface‐treated by sandblasting followed by 180 s of MMA application, then relined with autopolymerizing PMMA. Part 2: Forty‐five PLAD specimens were divided into three groups: PLAD (control), PLAD MC (methylene chloride treatment), and PLAD AT (acetone treatment), then relined with autopolymerizing PMMA. All samples underwent thermocycling before being evaluated for SBS with a universal testing machine (Shimadzu, Japan). Statistical analysis was performed using one‐way ANOVA and Tukey’s HSD at a significance level of *p* ≤ 0.05. Failure modes were examined using a stereomicroscope, and surface morphology was evaluated by scanning electron microscopy (SEM).

**Result:**

In Part 1, the mean SBS values in MPa were: PMAC (26.85 ± 1.31), PMAM (22.92 ± 1.20), PMAD (13.98 ± 1.20), PLAI (10.19 ± 1.59), and PLAD (8.44 ± 1.14). Significant differences were found between all groups (*p* ≤ 0.05). In Part 2, the mean SBS values in MPa were: PLAD (8.44 ± 1.14), PLAD MC (8.35 ± 0.84), and PLAD AT (7.83 ± 1.13). No significant differences were found between the three groups (*p*  > 0.05). PMAC, PMAM, and PMAD exhibited mixed failure modes in 87.5%, 80%, and 73.3% at the fractured surfaces of the specimens, respectively. In contrast, PLAD and PLAI displayed 100% adhesive failure. PLAD, PLAD MC, and PLAD AT also showed 100% adhesive failure at fractured surfaces.

**Conclusion:**

The PMAC group achieved the highest SBS among all tested materials. Surface treatment of PLAD with methylene chloride or acetone did not significantly improve SBS compared to the untreated control.

## 1. Introduction

Provisional crowns are essential components of fixed prosthodontic treatment, serving to protect the dental pulp, maintain periodontal health, and preserve esthetics during the interim phase before final restoration. Autopolymerizing polymethyl methacrylate (PMMA) remains the conventional standard due to its favorable mechanical properties and cost‐effectiveness. However, its exothermic polymerization and dimensional changes may compromise marginal adaptation and potentially irritate oral tissues [1].

Contemporary dentistry increasingly utilizes computer‐aided design and computer‐aided manufacturing (CAD/CAM) systems for fabricating provisional crowns. This digital workflow, encompassing subtractive manufacturing (milling) and additive manufacturing (3D printing), offers advantages such as reduced chair time, improved accuracy, and decreased waste [2, 3]. Digital provisional crowns often exhibit superior mechanical properties, including fracture strength and wear resistance, compared to conventional PMMA [4], thereby making them suitable for long‐term temporization.

Beyond conventional autopolymerizing PMMA, alternative materials such as polylactic acid (PLA) have emerged for provisional crown fabrication. PLA is a widely used polymer derived from renewable, nontoxic sources, including corn, wheat, sugar, and other crops, through an environmentally friendly process that utilizes atmospheric carbon dioxide. The most notable feature of PLA is its excellent biocompatibility, particularly for biomedical applications. PLA produces no toxic effects, its degradation byproducts do not interfere with tissue healing, and it demonstrates superior thermal processability for various manufacturing methods, including extrusion, injection molding, blow molding, fiber spinning, and thermoforming [5].

The structure of PLA belongs to the family of poly‐α‐hydroxy acids, classified as a linear aliphatic thermoplastic polyester. This material exhibits favorable mechanical properties, elastic modulus, and thermoforming characteristics, with minimal deformation during printing due to its low shrinkage rate [6]. PLA is commonly employed in additive manufacturing techniques, particularly fused deposition modeling (FDM), where it provides good dimensional stability, ensuring accurate and precise prints [7]. Its versatile properties, including low cost, biocompatibility, processability, and mechanical strength, make it a promising polymer for applications in the human body [8].

The FDA has approved PLA for several medical applications, including absorbable sutures and orthopedic screws [9]. For dental applications, PLA materials for provisional crowns have received approval from the Korea Food and Drug Association (KFDA) [10]. In vitro studies have demonstrated acceptable fit, mechanical strength, and biocompatibility for temporary crowns [11]. Therefore, in the near future, PLA may become a viable material and widely used for fabricating provisional crowns in clinical practice.

Clinical provisional crowns often require a relining procedure, typically using autopolymerizing PMMA, to enhance adaptation and improve marginal fit. Research has established that materials with identical chemical structures (e.g., PMMA to PMMA) demonstrate better adhesion than those with dissimilar structures [12].

However, with current digital technology advancing the creation of provisional crowns, it is crucial to identify alternative materials that can achieve comparable properties to conventional relining methods. The performance of newer materials such as PLA, produced through injection or 3D printing, when reinforced with autopolymerizing PMMA requires investigation. If these materials possess comparable or lower properties than the conventional method, surface treatment with various materials may enhance retention. A prior study found that combining sandblasting or hydrofluoric acid etching with priming is essential for achieving optimal bonding between self‐adhesive resin cement and CAD/CAM resin composites. This information is valuable for dental professionals during restorative procedures, and further research on long‐term performance is recommended [13]. Various solvent‐based materials may provide surface modification effects, including methylene chloride or acetone. Methylene chloride is the same substance as dichloromethane, an organochlorine chemical with the formula CH_2_Cl_2_. This colorless, volatile liquid with a chloroform‐like, pleasant odor is commonly employed as a solvent. Although it is not miscible with water, it is mildly polar and compatible with a wide range of organic solvents [14]. Acetone, also known as dimethyl ketone, is an organic chemical with the molecular formula (CH_3_)_2_CO. This is the simplest and smallest ketone (R─C(═ O)─R’). It’s a colorless, extremely volatile, and combustible liquid with a distinctive pungent odor. Acetone is miscible with water and widely used as an organic solvent in business [15].

Therefore, the objective of this study is to compare the shear bond strengths (SBSs) between different types of provisional crown materials (conventional, milled, and 3D‐printed PMMA, as well as injected and 3D‐printed PLA) and various solvent‐based surface treatments when relined with autopolymerizing PMMA.

This study hypothesizes that there will be no statistically significant difference in the SBS among different types of provisional crown materials (PMAC, PMAM, PMAD, PLAI, and PLAD) when relined with autopolymerizing PMMA. The surface treatment of 3D‐printed PLA (PLAD) with methylene chloride or acetone will show no statistically significant difference in the SBS compared to the untreated control group when relined with autopolymerizing PMMA.

## 2. Materials and Methods

### 2.1. Specimen Preparation

#### 2.1.1. Experimental Part 1

The different types of provisional crown materials, divided into five groups, each with 15 specimens. The specimens were 10 mm in diameter and 10 mm in height. The materials used are shown in Table 1.

**Table 1:** Materials used in Experimental 1.

Group	Material	Composition	Manufacturer
PMAC	Conventional autopolymerizing PMMA	Polymethyl methacrylate, benzoyl peroxide, methy methacrylate monomer, tertiary amine	Unifast Trad GC America Inc., Alsip, IL, USA
PMAM	Milling VIPI Block Trilux multilayer PMMA	Polymethyl methacrylate, organically modified ceramics, EDMA	VIPI, Pirassununga, Brazil
PMAD	3D‐printed PMMA	Photopolymer resin, inorganic microfillers, hybrid composite structure	NextDent C&B MFH, Vertex‐Dental B.V., Soesterberg, The Netherlands
PLAI	Injection molding PLA	100%PLA, lubricant additives	Ingeo Biopolymer 3052D, NatureWorks LLC, Minnetonka, MN, USA
PLAD	3D‐printed PLA	92%–96%PLA, 2%–4% calcium carbonate, 2%–5% impact modifiers and additives	eSUN PLA, Shenzhen, China

**Figure 1:**
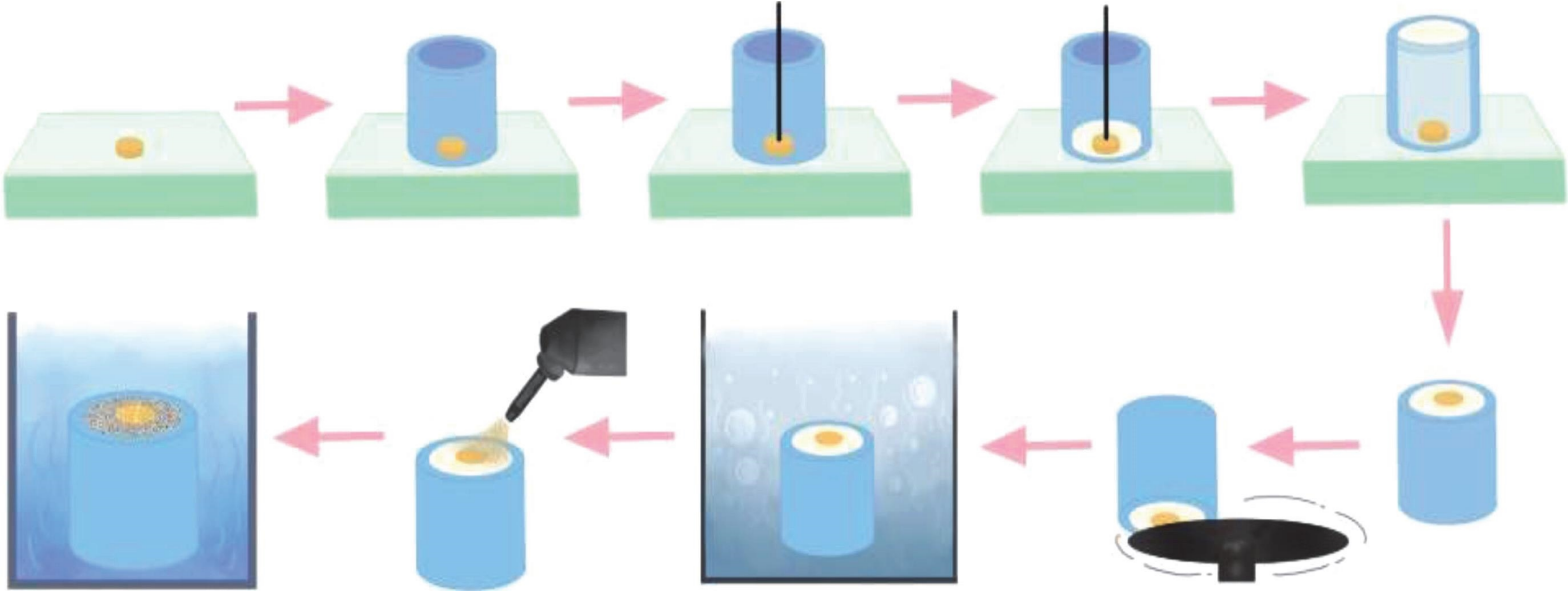
Illustration of the experimental procedure.

All the specimens were placed in 22 mm diameter polyvinyl chloride tubes with an internal diameter of 18 mm and 3 cm in height and bonded with self‐cured acrylic resin (Formatray, Germany, Maydent). The surfaces of embedded specimens were polished with 600‐ and 800‐grid abrasives (TOA, Thailand) using a polishing machine (EcoMet 30 Semi‐Automatic Grinder‐Polisher, Buehler, USA) for 15 s. Then, cleansing in an ultrasonic bath (VGT‐19990QTD, China) for 15min, and air‐dried at room temperature. The specimens were subjected to sandblasting, using aluminum oxide particles (Al_2_O_3_, 50 μm, Hi‐ALUMINA, Shofu, Kyoto, Japan) in a sandblasting cabinet (Renfert, Germany) at an air pressure of 2 MPa for 15 s at a distance of 10 mm. They were cleaned under an ultrasonic bath with deionized water for 15 min and then soaked in distilled water for 24 h. All the procedures are shown in Figure 1.

**Figure 2:**
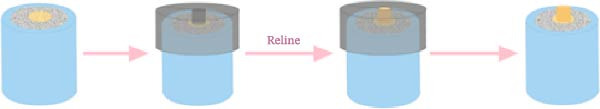
Relining procedure for specimen preparation.

After the surface preparation procedures, the specimens were applied with MMA (Unifast Trad, GC America Inc., USA) for 180 s. Then the specimens were covered with the metal mold, poured the reline material (Unifast Trad, GC America Inc., USA), which was mixed according to the manufacturer’s instructions into the center hole of the mold. The hole had a 5 mm diameter and a height. A load of 1000 g was placed on top of the metal mold for 5 min. The procedure is shown in Figure 2.

#### 2.1.2. Experimental Part II

The PLAD specimens (30 specimens) were divided into two groups, each group with 15 specimens. The materials used are shown in Table 2.

**Table 2:** Materials used in Experimental 2.

Group	Material	Composition	Manufacturer
PLAD MC	PLAD + methylene chloride	99.9% dichloromethane	Ngamsilp, Thailand
PLAD AT	PLAD + acetone	80% dimethyl ketone	CHEMRICH, Thailand

PLAD MC: the specimens were treated with methylene chloride (99.9% Dichloromethane, Ngamsilp, Thailand) for 180 s,

PLAD AT: the specimens were treated with acetone (80% dimethyl ketone, CHEMRICH, Thailand) for 30 s.

**Figure 3:**
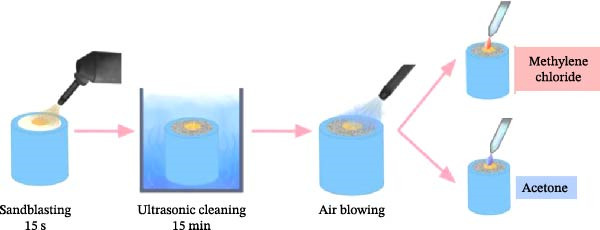
Methylene chloride and acetone surface treatments of PLAD specimens.

All procedures are shown in Figure 3. After applying MMA for 180 s, the specimens were covered with the metal mold, and the reline material was poured into the mold. A load of 1000 g was placed on top of the metal mold for 5 min.

All specimens from each group were subjected to thermocycling in two different thermal baths at 5°C and 55°C. Distilled water was used to maintain the temperatures of the thermal baths. Each sample was exposed to a thermocycling machine (Thermocycler 1100/1200, SD Mechatronik, Feldkirchen‐Westerham, Germany) for 20 s at 5 and 55°C with a 5 s interval between each cycle. A total of 500 cycles were carried out for each sample. Then, the specimens were immersed in distilled water for 24 h before the SBS test.

### 2.2. SBS Test

SBS testing was conducted using a universal testing machine (EZ‐s, Shimadzu, Japan). The sample was positioned horizontally with the assistance of a metal fixture. A metal blade with a thickness of 0.25 mm was moved vertically at the crosshead with a speed of 1 mm/min to break the bonding surface at the specimen surface and the PMMA interface. The maximum load at the break for each specimen was divided by the bonding area (mm^2^) to calculate the bond strength in MPa.

### 2.3. Evaluation Mode of Failure

After the SBS test, the de‐bonded specimens were analyzed with a stereomicroscope (SZ‐61, Olympus, Japan) using 15x magnification to grossly categorize the mode of failure into three types: adhesive failure, where the debonding occurred between the specimen and the relining material; cohesive failure, where the debonding occurred in the specimen or the PMMA reline. If both adhesive and cohesive failure contributed to the debonding, with at least 25% of each type in the same specimen, it was categorized as mixed failure.

### 2.4. Scanning Electron Microscope (SEM)

The specimens were sandblasted, applied with MMA, methylene chloride, and acetone, then inspected with 3000x magnification using SEM (JSM‐5410LV; JEOL, Tokyo, Japan).

## 3. Results

The experimental data were analyzed for normality with the Kolmogorov–Smirnov and Shapiro–Wilk tests. Then, the variance between each group was tested using the Levene test. If both tests indicated normal distribution, parametric tests could be performed. The obtained results were statistically analyzed (SPSS 25.0 for Macs; SPSS Inc., Chicago, IL) for comparison of the SBS using one‐way ANOVA. A post hoc test was then performed with Tukey HSD with *p* ≤ 0.05.

### 3.1. Experimental Part I

**Figure 4:**
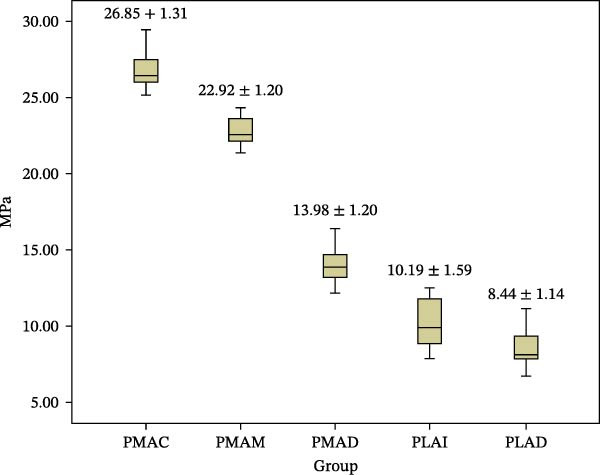
The mean SBS value and standard deviations of PMAC, PMAM, PMAD, PLAI, and PLAD.

The mean SBS values and standard deviations of all the experimental groups are summarized in Figure 4.

PMAC significantly demonstrated the highest SBS among the other groups. The bond strength value was followed by PMAM, PMAD, and PLAI, respectively, and the lowest bond strength was PLAD. Ultimately, all the experimental groups demonstrated a significant difference in SBS (*p* ≤ 0.05).

**Figure 5:**
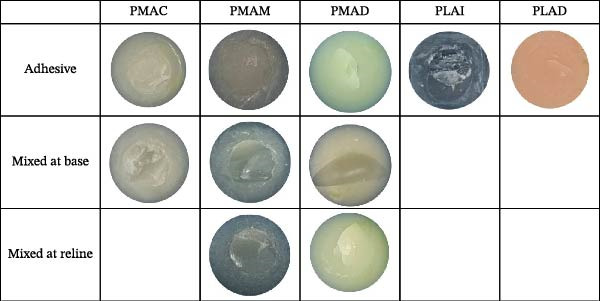
Examples of surface characteristics showing failure modes in specimens.

All the samples from various groups were analyzed for mode of failure using a stereomicroscope. The PMAC, PMAM, and PMAD showed mixed failure 87.5%, 80%, and 73.3% at the fractured surface of the specimens, respectively. On the other hand, the PLAD and PLAI groups displayed 100% adhesive failure. The result is shown in Table 3, and the example of the surface characteristics of the experimental specimens from each group demonstrates the mode of failure as shown in Figure 5.

**Table 3:** The number of specimens classified by the mode of failure.

Mode of failure	PMAC	PMAM	PMAD	PLAD	PLAI
Adhesive	3	4	8	15	15
Mixed (base)	12	4	5	0	0
Mixed (reline)	0	7	2	0	0

### 3.2. Experimental Part II

**Figure 6:**
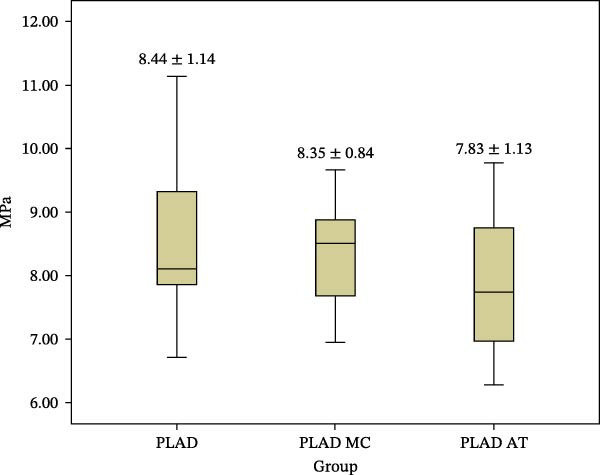
The mean SBS value and standard deviations of PLAD, PLAD MC, and PLAD AT.

The mean SBS values and standard deviations of the experimental groups PLAD, PLAD MC, and PLAD AT are presented in Figure 6.

According to the results of Experimental Part I, the PLAD group had the lowest SBS. To increase the SBS of this group, surface treatments with methylene chloride and acetone were applied. The results revealed no statistically significant differences in SBS among groups PLAD, PLAD MC, and PLAD AT (*p*  > 0.05).

**Figure 7:**
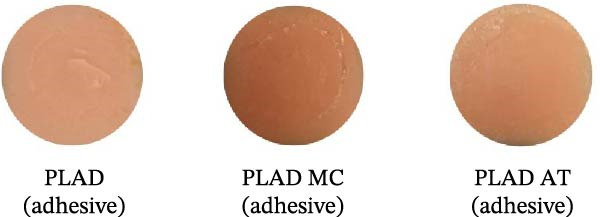
Examples of surface characteristics showing failure modes in PLAD, PLAD MC, and PLAD AT specimens.

The experimental samples from those groups were analyzed for failure mode using a stereomicroscope. The results showed similar failure patterns across all groups. Group PLAD, PLAD MC, and PLAD AT showed 100% adhesive failure at the fractured surface, as shown in Table 4. Surface characteristics demonstrating the failure modes for each group are shown in Figure 7.

**Table 4:** Number of specimens classified by mode of failure.

Mode of failure	PLAD	PLAD MC	PLAD AT
Adhesive	15	15	15
Mixed (base)	0	0	0
Mixed (reline)	0	0	0

**Figure 8:**
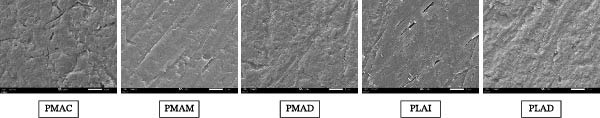
SEM images (3000× magnification) of PMAC, PMAM, PMAD, PLAI, and PLAD surfaces after sandblasting.

**Figure 9:**
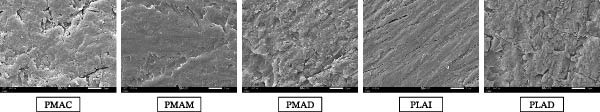
SEM images (3000× magnification) of PMAC, PMAM, PMAD, PLAI, and PLAD after sandblasting and applying MMA.

The surface characteristics of the experimental specimens, part I (PMAC, PMAM, PMAD, PLAI, and PLAD), were investigated using SEM at 3000x magnification. Images were obtained after sandblasting and applying MMA for 180 s, as shown in Figures 8 and 9.

**Figure 10:**
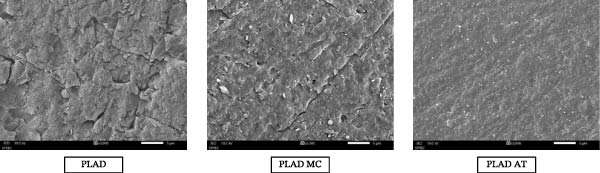
SEM images (3000× magnification) of PLAD, PLAD MC, and PLAD AT after sandblasting, treatment with MMA, methylene chloride, and acetone.

The surface characteristics of PLAD, PLAD MC, and PLAD AT were examined by SEM (3000x magnification) following sandblasting and chemical treatment with MMA, methylene chloride, and acetone, as demonstrated in Figure 10.

## 4. Discussion

This study evaluated the SBS of autopolymerizing PMMA and digitally fabricated provisional materials (PMMA and PLA) relined with the same autopolymerizing PMMA. The PMAC group demonstrated the highest SBS, supporting previous evidence that materials with similar chemical structures bond more effectively than those with dissimilar compositions [12]. This corresponds with the predominant mixed failure mode in PMAC, whereas PLA, showing the lowest SBS, exhibited exclusively adhesive failures. These findings align with earlier studies reporting higher SBS and mixed failure patterns in heat‐cured PMMA compared with 3D‐printed PMMA [16]. Similarly, relining 3D‐printed PMMA with PMMA has previously shown superior SBS compared with bis‐acryl and light‐cured materials [1]. The reduced bonding in 3D‐printed resins may be related to their bisphenol‐A dimethacrylate‐based chemistry, which limits MMA cross‐linking at the interface [17].

Thermocycling also influenced the bond strength of provisional materials. Water absorption during thermal cycling softens acrylic polymers and reduces mechanical stability, consistent with earlier findings in self‐cured PMMA [17]. PMMA bonding in PMAC may have been facilitated by similar water absorption behavior, while PLA exhibited lower water uptake but greater interfacial stress due to the mismatch in the coefficient of thermal expansion (CTE) between PLA and PMMA, contributing to reduced SBS and adhesive failures [18].

Mechanical surface treatment enhanced surface roughness and micromechanical retention, as confirmed by SEM. Previous work has identified Al_2_O_3_ sandblasting as producing the highest roughness and bond strength [19]. In a recent study, Taokhampu et al. [20] investigated how well repair materials bond to aged 3D‐printed provisional resins after different surface treatments. They found that the way the surface was prepared had a major impact on bond strength. Air abrasion with Al_2_O_3_, whether used alone or followed by adhesive, produced the strongest bonds. In contrast, surfaces that were not treated at all showed the weakest bonding. These findings suggest that for aged 3D‐printed provisional resins that have been creating a rough surface and applying adhesive are essential steps for achieving successful repairs [20].

Chemical surface treatment with MMA further improved wetting and penetration of the reline resin, consistent with earlier reports [21]. Relining the provisional crown with autopolymerizing PMMA shows that pretreating with a monomer enhances bonding between the materials. This phenomenon is supported by the increased surface swelling compared to earlier, as shown in the SEM Figure 9.

The PLAD group displayed the lowest SBS in Experimental Part I, so Experimental Part II was conducted to investigate methods for enhancing bond strength. Organic solvents such as chloroform, acetone, and methylene chloride can be used for chemical surface treatment. The prior research reported that these organic solvents increase the bond strength of repair materials to the denture base [22]. However, for this research, the chemical surface treatment in Groups PLAD with methylene chloride and acetone was not statistically significant. Lavecchia et al. [23] immersed PLA specimens in acetone for 180 s, and the bonding improved significantly. Chladek et al. [24] applied PLA with MMA, ethyl acetate, isopropyl alcohol, and methylene chloride for 30 s and achieved similar results. FTIR analysis showed that acetone chemically modified the PLA surface through partial hydrolysis, creating polar groups that improved wettability and adhesive bonding. Conversely, mechanical abrasion physically roughened the surface, improving adhesion through mechanical interlocking, as confirmed by fracture analysis [25].

In our study, the specimens of Group PLAD were treated with different chemicals: methylene chloride for 180 s and acetone for 30 s; the differences in bonding strength were not statistically significant. The experimental procedure may have influenced results because mechanical surface treatment preceded chemical treatment. Sandblasting creates a relatively deep surface roughness, which reduces the effectiveness of chemical surface treatment. When observing the mode of failure before and after chemical surface treatment, it is evident that the failure mode remains the same, which is the adhesive.

SEM observations showed deeper pits in PMAC, PMAM, and PMAD than in PLA groups following sandblasting. Although MMA and acetone produced surface swelling and texture changes in PLA during chemical treatment, these morphological alterations did not translate into higher SBS.

## 5. Conclusion

Among the tested groups in these experiments, the conventional autopolymerizing PMMA group achieved the highest shear bond strength when relined with autopolymerizing PMMA. For PLA 3D‐printed materials, surface treatment with methylene chloride or acetone following sandblasting did not produce statistically significant differences in shear bond strength when relined with autopolymerizing PMMA.

## Funding

This work was supported by the Research Institute of Rangsit University (Grant 53/2566).

## Conflicts of Interest

The authors declare no conflicts of interest.

## Data Availability

The data that support the findings of this study are available from the corresponding author upon reasonable request.
